# Coronavirus disease 2019 (COVID-19) personal protective equipment training: Using simulation-based training to prevent healthcare-associated infection

**DOI:** 10.1017/ash.2022.23

**Published:** 2022-02-18

**Authors:** Madhuri B. Nagaraj, Krystle K. Campbell, Minji Kang, Ian A. Nazareno, Doramarie Arocha, Julie B. Trivedi, Daniel J. Scott

**Affiliations:** 1 Simulation Center, University of Texas Southwestern Medical Center, Dallas, Texas; 2 Division of Infectious Diseases and Geographic Medicine, Department of Medicine, University of Texas Southwestern Medical Center, Dallas, Texas; 3 Infection Prevention, University of Texas Southwestern Medical Center, Dallas, Texas

## Abstract

In this pre- and postintervention study, we demonstrate the feasibility and effectiveness of comprehensive simulation-based personal protective equipment (PPE) training amid the COVID-19 pandemic. With rapid-cycle, deliberate practice feedback, simulation-based training can improve the learners’ sense of confidence and security while standardizing PPE protocols.

Simulation-based training (SBT) has served as an important adjunct to traditional teaching methods in healthcare education. Among its many roles, SBT has allowed standardization of practice, allowing learners to acquire procedural competence through repeated practice and feedback in an interactive environment. The self-contamination rate can be as high as 50% in the use of personal protective equipment (PPE),^1^ and simulation-based PPE training has proven to be efficacious in outbreaks such as Ebola and severe acute respiratory syndrome.^2^ With the need for proper implementation of PPE guidelines during the coronavirus disease 2019 (COVID-19) pandemic, we identified the need for PPE training of our frontline staff. In this study, we assessed the feasibility of comprehensive simulation-based PPE training amid the COVID-19 pandemic while measuring its impact on learners’ comfort and anxiety around COVID-19 patient care.

## Methods

### Study setting

The University of Texas Southwestern Medical Center (UTSW) is a large, public, academic, health science center. The UTSW Simulation Center, in partnership with infection prevention, nursing education, emergency preparedness, and campus leadership, led a campus-wide initiative for COVID-19 PPE training at the start of the pandemic. An interprofessional, simulation-based, blended-theory program grounded in experiential learning was implemented for healthcare personnel (HCP) across multiple departments.

### Intervention

Content and training materials were built from the best-practice guidelines from the Centers for Disease Control and Prevention^3^ and National Emerging Pathogens Preparedness training center.^4^ These materials were vetted by local infection prevention and education experts. The instructional design components used experiential theory and included (1) an asynchronous online module with videos, checklists, and a narrated presentation reviewing the critical techniques of donning/doffing PPE and purified air powered respirators (PAPRs) as well as (2) an in-person SBT that provided expert demonstration with equipment and formative feedback on learners’ skills. The center deployed a train-the-trainer model to recruit a multiprofessional team of educators to lead in-person sessions. Each in-person session consisted of 1 trainer to 4 learners to allow for adequate social distancing. Learners received rapid-cycle, deliberate practice feedback until they reached a level of competence at which they effectively executed 80% of the donning and doffing checklist.

### Pre- and postintervention surveys

Pre- and postintervention surveys were administered during in-person sessions to assess the learners’ history of simulation training, previous experience with PPE, pre- and postintervention comfort with PPE, effectiveness of the educator, and usefulness of the training tool. The questions were scored using a Likert scale from 1 (not at all) to 4 (very much). Comfort and preparedness were defined as learners reporting a 3 (moderately) or 4 (very much) to the respective questions. Anxiety levels surrounding COVID-19 patient care were assessed with the validated State Trait Anxiety Inventory (STAI)-6 questionnaire.^5^ Scores ranged from 20 to 80, with higher scores indicating increased psychological stress.

Data were analyzed using SPSS version 25 software (IBM, Armonk, NY). Categorical data were reported as proportions and analyzed with χ^2^ tests. Continuous variables were reported as means and standard deviations (SD), and the Student *t* test was used to analyze differences between means. All tests were 2-sided and *P* < .05 was considered statistically significant.

## Results

### Demographics

From July 1 to December 31, 2020, 1,918 learners enrolled in simulation-based PPE training and 1,873 completed the full training. Over 740 hours of training, 895 (48%) medical students, 425 (23%) residents, 139 (7%) faculty, and 108 (6%) nurses were trained (Table 1). The 26 trainers comprised 10 allied health professionals, 7 nurses, 6 simulation center staff, 2 fellows, and 1 faculty member.

**Table 1.** Learner Type for COVID 19 Personal Protective Equipment Simulation-Based Training

Learner Type	Total (N = 1,873), No. (%)
Medical students	895 (48)
Residents	425 (23)
Faculty	139 (7)
Registered nurse	108 (6)
Physician assistant students	105 (6)
Fellows	98 (5)
Phlebotomist	26 (1)
Physical therapist	23 (1)
Patient care technician	20 (1)
Occupational therapist	14 (<1)
Therapy technician	9 (<1)
Physician assistant	6 (<1)
Specimen processor	2 (<1)
Mental health technician	1 (<1)
Polysomnographer	1 (<1)
Speech language pathologist	1 (<1)

### Survey response

Overall, 795 individuals completed the preintervention survey and 848 completed the postintervention survey. When asked about previous PPE experience, 77% had experience wearing PPE, 34% had formal PPE training, and 6% had prior experience with PAPR. Significant increases in comfort in donning and doffing PPE and PAPR were reported across all learner groups: donning PPE (64% before the intervention versus 98% after the intervention; *P* < .01), doffing PPE (61% versus 99%; *P* < .01), donning PAPR (18% versus 40%; *P* < .01), and doffing PAPR (18% vs 40%; *P* < .01) (Table 2). In aggregate, 95% of learners felt that the curriculum made them prepared for PPE usage to care for COVID-19 patients and 93% felt that the hands-on practice was worthwhile. When asked whether learners would participate in future simulation experiences, there was a significant increase after the training (44% vs 69%; *P* < .01).

**Table 2.** Pre- and Postintervention Survey

Survey Question	Preintervention Survey (N = 795), Mean (±SD)	Postintervention Survey (N = 848), Mean (±SD)	*P* Value
I feel comfortable donning PPE	2.84 (± 0.04)	3.76 (± 0.48)	<.01
I feel comfortable doffing PPE	2.79 (± 0.88)	3.74 (± 0.50)	<.01
I feel comfortable donning a PAPR	1.59 (± 0.95)	2.22 (± 1.25)	<.01
I feel comfortable doffing a PAPR	1.58 (± 0.94)	2.21 (± 1.25)	<.01

Note. SD, standard deviation; PPE, personal protective equipment; PAPR, purified air-powered respirators.

In response to the STAI-6 anxiety scale, there was no significant change (46.0 before training vs 46.6 after training; *P* = .385), and 87% of learners categorized themselves to have “moderate” or “high” anxiety. The “high anxiety” category increased by 8.6% (*P* < .01) after the intervention. Our secondary analysis demonstrated this trend to be driven by physicians, with a 3.6% increase (*P* < .01), and medical students, with a 4.3% increase (*P* = .023).

## Discussion

Our study demonstrates the effectiveness of comprehensive simulation-based PPE training across various disciplines amid the COVID-19 pandemic. With limited availability of infection preventionists due to competing responsibilities in the COVID-19 pandemic, we implemented a train-the-trainer model and recruited reassigned nurses, residents, and health educators to serve as instructors. We kept learners and instructors safe by using small class sizes that allowed for social distancing and simultaneously held 4 sessions per hour to accommodate the large number of learners who required training. Due to PPE shortages, we created low-fidelity N95 respirators and utilized reusable gowns that were sanitized between sessions to overcome supply-chain challenges while maintaining the learning experience.

Our results reaffirm the overall insufficient training in infection prevention; only 34% of learners reported prior history of PPE training. This finding is consistent with prior studies demonstrating the lack of formal PPE training or assessment of proficiency among HCP across multiple disciplines.^6,7^ HCP train on the job rather than through standardized group training or formal teaching sessions. Such inadequacy in PPE training is demonstrated in high self-contamination rates while doffing PPE.^1^ SBT can potentially close this gap; our survey results demonstrate an increase in comfort in donning and doffing PPE and PAPRs. Interestingly, we did not witness any improvement in anxiety scores, with most remaining in the “moderate” to “high anxiety” categories before and after the intervention. Possibly, exposing a lack of knowledge or experience with COVID-19 led to the increase in anxiety, but further work is needed to determine whether repeated training and desensitization could improve anxiety metrics.

This study had several limitations. This study was performed in a single institution, which may limit the generalizability of our PPE training. The UTSW has a dedicated simulation center and staff, which may not be available at other institutions. However, given the use of inexpensive, low-fidelity materials and sustainable train-the-trainer teaching approach, our efforts are scalable and can be reproduced at other institutions. In addition, no validation measures were performed for the pre- and postintervention surveys. Although the pre- and postintervention survey results demonstrated increase in comfort, we had no data on compliance in clinical environment or its impact on healthcare-acquired COVID-19. Finally, we did not assess knowledge retention or the need for future sessions.

By identifying missteps and knowledge gaps, SBT served as an effective training modality that significantly increased the learners’ sense of confidence and security while standardizing PPE protocols. This study has demonstrated the benefits of SBT and the need for continued innovation to ultimately improve competence and compliance of infection prevention strategies.
